# Italian family paediatricians’ approach and management of celiac disease: a cross-sectional study in Puglia Region, 2012

**DOI:** 10.1186/1471-230X-14-38

**Published:** 2014-02-20

**Authors:** Francesca Fortunato, Domenico Martinelli, Vanessa Cozza, Pierpaolo Ciavarella, Anna Valente, Teresa Cazzato, Ruggiero Piazzolla, Rosa Prato, Biagio Pedalino

**Affiliations:** 1 Department of Medical and Surgical Sciences, University of Foggia, Viale Pinto, 71100 Foggia, Italy; 2 Representatives of the Apulian Federazione Italiana Medici Pediatri (FIMP), Via S. Antonio 73, 70051 Barletta, Italy

**Keywords:** Celiac disease, Knowledge attitude and practice, Paediatricians

## Abstract

**Background:**

Celiac disease (CD) is a chronic autoimmune illness of the small intestine triggered by gluten consumption in genetically predisposed individuals. CD presentation is not limited to the gastrointestinal tract and it is still under-diagnosed. Complete resolution of clinical manifestations follows if a gluten-free diet is adopted. In western countries, CD prevalence is approximately 1%. Age of onset is often between 6 months and 7 years.

We assessed the approach to diagnosis and management of celiac patients by the paediatricians in Puglia Region, Italy.

**Methods:**

We conducted a cross-sectional survey among the 589 Apulian Family Paediatricians (FPs) during January 2011-January 2012 using a self-administered web-based standardized questionnaire including self-assessment of their knowledge, diagnostic path and type of management they would follow for CD, clinical information on their celiac patients. We assessed associations among the explored variables by defining double-entry contingency tables and calculating Odds Ratio (OR) with 95% Confidence Intervals (CIs).

**Results:**

The 218 (37%) FPs participating in the study reported 1,020 CD patients (representing approximately 1% of the child population covered by the enrolled FPs). Of them, 55% were female; 45% were aged 5–10 years. Weight loss and stunting were the main reported symptoms at diagnosis (41%). The majority (98%) of FPs requested anti-transglutaminase antibody (tTG-Ab) titres for CD diagnosis. Approximately 78% of FPs recommended gluten introduction in the diet of infants at the age of 6 months; 12% and 8% recommended introduction of gluten before and after 6 months of age respectively.

The degree of knowledge for either CD diagnosis making process or CD related diseases was medium/high in 97% and 82% of the participating FPs respectively. FPs (83%) who had a medium or high degree of knowledge of CD patients’ diet were more likely to experience low or no difficulty in providing their patients with dietary advices (OR:5.5; 95%CI:1.7-17.5).

**Conclusions:**

Apulian FPs report a good degree of knowledge of CD, its diagnosis and its management. We will diffuse results and recommendations to all paediatricians in the Region. Actions aiming to continued education on CD in medical under and postgraduate trainings are crucial to prevent under-diagnosis.

## Background

Celiac disease (CD) is a chronic autoimmune illness of the small intestine triggered by gluten consumption in genetically predisposed individuals. In these individuals, exposure to gluten produces mucosal damage that, following different stages of disease severity, may result in small-intestinal mucosal atrophy. CD clinical manifestations are not limited to the gastrointestinal tract and systemic signs can be commonly associated (i.e. anemia, osteoporosis, short stature, arthritis, infertility, peripheral neuropathy and dermatitis herpetiformis) [1-5]. A complete resolution of the clinical manifestations and of the intestinal mucosal lesions is obtained if a gluten-free diet is adopted [6]. However, CD is still heavily underdiagnosed, approximately 75-90% of the celiac population in western countries remains unrecognized, presumably due to the absence, or the atypical nature of symptoms, but also as a consequence of the poor physician awareness of the clinical spectrum of the disease [7,8].

In western countries, the prevalence of CD in the general population is approximately 1% and the female:male ratio of 2:1 [1,2,7,9,10]. Recent US and European studies show however a 2–4.5 fold increase in the disease prevalence [11-13]. In Italy, CD prevalence is between 0.55% and 1% [14].

The age of onset of CD is often between 6 months and 7 years (the median age when CD antibody markers develop is 3 years) [15]. Prevalence is higher among risk patients, i.e. type 1 diabetes mellitus (varying from 3 to 6%) and first-degree relatives of celiac patients (up to 20%) [16].

Anti-transglutaminase Antibody (tTG-Ab) and Endomysial Antibody (EMA-Ab) are the most frequently used serological tests although Anti-gliadin Antibody (AGA-Ab) measurement is also available. In dubious cases, genetic testing is now used to exclude diagnosis [17]. According to the European Society of Paediatric Gastroenterology, Hepatology, and Nutrition (ESPGHAN) guidelines, a control biopsy is considered mandatory for asymptomatic patients at first presentation or those with equivocal response to the diet to verify the effects of the gluten-free diet on the architecture of the intestinal mucosa. Gluten challenge is advisable when the initial diagnosis or the clinical response to a gluten-free diet is uncertain. Furthermore, the ESPGHAN guidelines recommend gluten challenge, preceded by HLA typing and assessment of mucosal histology and discouraged before the child is 5 years old [17,18]. According to ESPGHAN and EFSA (European Food Safety Authority) introduction of solid foods in the diet of infants before the end of the 3rd month of life should be avoided as children might develop food allergies [19-24].

In Italy, by law, every child is included in the list of patients of a Family Paediatrician (FP) from birth until the age of fourteen years. In case of specific illnesses or disabilities, assistance may be extended up to the age of 16 years. Normally, the number of children included in the list of the FPs is known to the public and can reach a maximum of 800 children; a number of exceptions are accepted, i.e. lack of paediatricians in the territory, a new child in a family with other children followed up by the same paediatrician [25,26]. FPs’ role is to follow up their patients in terms of regular check up and monitor their growth, provide advices for the maintenance of their health wellbeing, suggest the necessary diagnostic tests to identify diseases, prescribe proper treatment and support families for the best management of their children chronic diseases [27].

We described the knowledge of CD, approach to diagnosis and management of the celiac patient by the paediatricians in Puglia Region, Italy.

## Methods

We conducted a cross-sectional study among the 589 Apulian FPs during the period January 2011 - January 2012. We invited all FPs by email sending them an explanatory letter introducing the study and its objectives and the link to a self-administered web-based standardized questionnaire (http://www.surveymonkey.com) including demographics and province of work of the paediatricians, clinical information on their celiac patients (i.e. clinical presentation at diagnosis), self-assessment of their knowledge, attitude, diagnostic path and type of management they would follow for CD. We sent weekly reminders to non-respondents.

We assessed the possible associations among the explored variables by defining double-entry contingency tables and calculating Odds Ratio (OR) with 95% CIs. The assessment of significant differences across the means of continuous variables relied on the *t*-test for independent samples considering as significant those values with p < 0.05. We used STATA-MP software, version 10.1 for Mac OS X.

### Ethics statement

The study received the approval by the Institutional Review Board of the Puglia (Italy) Regional Observatory for Epidemiology and was promoted by the regional division of the Italian Federation of Paediatricians. The study was conducted in accordance with Guideline for Good Clinical Practice and the ethical principles that have their origins in the Declaration of Helsinki. All participants were explained the purpose of the investigation and participation was voluntary and provided informed consent by e-mail.

## Results

Of the 589 FPs operating in the Region, 218 (37%) participated in the study (covering approximately 185,000 children); 7 FPs expressively refused to participate and 3 reported not to have any celiac patients, hence did not complete the questionnaire. Of the 218 respondents FPs, 57.3% were female, their median age was 54 years (range: 44–68; 1°quartile: 50; 3°quartile: 57) and they were from all the provinces of Puglia Region.

A total of 1,020 CD patients (representing approximately 1% of the children population covered by the participating FPs) were reported (an average of 5 ± 3 celiac patients per paediatrician). Of them, 55% were female; the mean of female patients for FP was higher than the male (2.5 ± 2.1 vs 1.5 ± 1.8; p < 0.0001); 45% of patients were aged from 5 to 10 years. Weight loss and stunting were the main reported symptoms at diagnosis (41%), followed by anaemia (22%), abdominal pain (19%) and chronic diarrhoea (17% - Table 1).

**Table 1:** Distribution of celiac patients by sex, age group and clinical presentation at diagnosis, Puglia Region, 2012

**Characteristic**	**Number**	**%**
**Sex**		
Female	556	54.5
Male	324	31.8
Missing	140	13.7
**Age group**		
0-1 year	16	1.6
2-4 year	201	19.7
5-10 year	457	44.8
11-16 year	206	20.2
Missing	140	13.7
**Clinical presentation at diagnosis**		
Weight loss and stunting	418	41.0
Anaemia	225	22.1
Recurrent abdominal pain	198	19.4
Chronic diarrhoea	170	16.7
Short stature	124	12.2
Asthenia	47	4.6
Hyper-transaminasemia	43	4.2
Constipation	41	4.0
Other symptoms	122	12

Among the participating FPs, 90% were those who first suspected CD in their patients. The vast majority (98%) of FPs requested anti-tTG titres for the diagnosis of CD; anti-EMA and anti-AGA titres were also requested (Table 2). Children were referred to a University Hospital (53%), to a public hospital (44%), and to another family paediatrician (3%) for CD diagnosis. FPs that normally referred to a public hospital, reported a medium-high level difficulty in the diagnosis making process when programming for a biopsy for their patients compared to those FPs that referred to a University Hospital that reported no or a low level difficulty (p < 0.0001).

**Table 2:** Confirmatory tests for celiac disease required by family paediatricians, Puglia Region, 2012

**Confirmatory test for celiac disease**	**%**
Anti-tissue transglutaminase antibody	98.2
Anti-endomysium antibody	77.5
Anti-gliadin antibody	61.0
Bowel biopsy	25.2
HLA DQ2 and DQ8	4.6
Other tests	17.4

Of the 215 (99%) responding to the question, 171 (78%) FPs recommended gluten introduction in the diet of infants at the age of 6 months; 27 (12%) and 17 (8%) recommended introduction of gluten before and after 6 months of age respectively.

The number of celiac patients was higher among those FPs that recommended gluten introduction in the diet after 6 months of age compared to those who would recommend the introduction of gluten between 4 and 6 months of age (4.1 ± 3.2 vs 3.3 ± 2.6, p = 0.1383).

The degree of knowledge for either CD diagnosis making process or CD related diseases was medium/high in 97% and 82% of the participating FPs respectively while it was low (37%) for CD related psychological disorders (Table 3).

**Table 3:** FPs self-reported degree of knowledge of celiac disease, Puglia Region, 2012

	**Degree of knowledge**				
	**None**	**Low**	**Medium**	**High**	**Missing**
	N (%)	N (%)	N (%)	N (%)	N (%)
CD diagnosis	0 (/)	3 (1.4)	125 (57.3)	87 (39.9)	3 (1.4)
Celiac diet	1 (0.5)	17 (7.8)	152 (69.7)	17 (7.8)	2 (0.9)
CD psychological disorders	10 (4.6)	80 (36.7)	99 (45.4)	21 (9.6)	8 (3.7)
CD related diseases	6 (2.8)	26 (11.9)	151 (69.3)	28 (12.8)	7 (3.2)

Almost 52% of the FPs were sometimes or often consulted for CD related diseases, followed by request of advices on CD related diet (39% - Table 4).

**Table 4:** FPs self-reported frequency of consultation for diet, psychological problems and diseases related to CD, Puglia Region, 2012

	**Frequency of consultation**				
	**Never**	**Rarely**	**Sometimes**	**Often**	**Missing**
	N (%)	N (%)	N (%)	N (%)	N (%)
**Diet**	58 (26.6)	68 (31.2)	68 (31.2)	17 (7.8)	7 (3.2)
**Psychological problems**	77 (35.3)	59 (27.1)	60 (27.5)	15 (6.9)	7 (3.2)
**Related diseases**	48 (22)	45 (20.6)	86 (39.4)	27 (12.4)	12 (5.5)

The participating FPs had medium or no difficulty to provide their patients with dietary advice (~75%), communication and information on CD (~66%), and to follow their patients up (~ 72%) (Table 5).

**Table 5:** FPs self-reported level of difficulty for programming a biopsy, for communication and information, and for dietary advice and any control or follow-up, Puglia Region, 2012

	**Level of difficulty**				
	**High difficulty**	**Medium difficulty**	**Low difficulty**	**No difficulty**	**Missing**
	N (%)	N (%)	N (%)	N (%)	N (%)
**Programming a biopsy**	51 (23.4)	65 (29.8)	43 (19.7)	58 (26.6)	1 (0.5)
**Communication and information**	10 (4.6)	55 (25.2)	70 (32.1)	73 (33.5)	10 (4.6)
**Dietary advice**	2 (0.9)	39 (17.9)	77 (35.3)	88 (40.4)	12 (5.5)
**Control or follow-up**	3 (1.4)	46 (21.1)	74 (33.9)	84 (38.5)	11 (5)

FPs (83%) who had a medium or high degree of knowledge of the diet for CD patients were more likely to experience low or no difficulty in providing their patients with dietary advices (OR: 5.5, 95% CI: 1.7-17.5; p < 0.001 – Table 6), and those (58%) who had medium or high degree of knowledge of CD related diseases were sometimes or often consulted for these disease (OR: 2.2, 95% CI: 0.9-5.2 p < 0.05 – Table 7).

**Table 6:** Association (OR with 95% CI) between self-reported degree of knowledge and level of difficulty on providing advices on CD diet among the participating FPs, Puglia Region, 2012

	**Degree of knowledge CD diet**					
	**None/low**		**Medium/high**		**OR (95% CI)**	***X*** **2**
**Level of difficulty advices on CD diet**	**N**	**%**	**N**	**%**		
**No/low difficulty**	8	47.1	156	83.0	5.5 (1.7-17.5)	12.6
**Most/medium difficulty**	9	52.9	32	17.0		
**Total**	17	-	188	-		

**Table 7:** Association (OR with 95% CI) between self-reported degree of knowledge and frequency of consultation CD related diseases among the participating FPs, Puglia Region, 2012

	**Degree of knowledge CD related diseases**					
	**None/low**		**Medium/high**		**OR (95% CI)**	***X*** **2**
**Frequency of consultation CD related diseases**	**N**	**%**	**N**	**%**		
**Never/rarely**	19	61.2	72	42.1	2.2 (0.9-5.2)	3.9
**Sometimes/often**	12	38.7	99	57.9		
**Total**	31	-	171	-		

More than 60% of the FPs reported themselves as the reference point for the celiac patient, 34% partly, and 4% did not considered themselves as reference point.

Approximately 90% of the FPs defined useful to receive regular update on the disease and 12% of FPs reported to receive updates on gluten-free diet by the sales representatives.

## Discussion

This is the first study evaluating the approach to diagnosis and management of celiac patient by family paediatricians in Italy.

Our findings show that FPs report a good degree of knowledge of CD related diseases and of gluten-free diet, although only a small proportion of them reported feeling comfortable with managing psychological disorders related to CD.

The prevalence of CD disease in our sample was consistent with other studies as well as the female to male ratio [1,2,7,9,10].

Similarly to other Italian studies, FPs participating in our survey were more inclined to comply with the guidelines that recommend anti-tTG-Ab measurement as the elective screening test for patients with a suspect of CD [17]. The vast majority of our paediatricians requested this test when suspecting celiac disease from the clinical symptoms.

The diagnosis of CD is important not only in children with obvious gastrointestinal symptoms but also in children with a less clear clinical picture due to the progressive worsening of the health conditions and long-term sequelae [15,28]. In our study, most paediatricians were the first to have an early suspect of CD in their patients although they were probably missing the CD diagnosis in some children with atypical symptoms as the proportion of children typical symptoms was higher than what has been shown in screening studies.

Most of the FPs participating in the study would suggest the introduction of gluten in the diet between 4 and 6 months of age (reducing, possibly, the occurrence of CD), similarly than in other countries where strong evidences suggest that solid food-items (including gluten-containing food) should be introduced in this age window [19,21,29]. Moreover, although not significant, a higher number of celiac patients was reported by those FPs who would suggest a gluten-containing diet after the age of 6 months. A higher risk of CD is in fact shown among children who had been introduced with gluten in the diet in the first 3 months or after the 7th month of life compared with introducing gluten in the 4- to 6-month period [30].

The study showed the paramount role of family paediatricians in the identification and diagnosis of CD among their patients. Childhood is a challenging period for diagnosis and management of CD considering that the growth of the child and the development of the immune system is an on-going process. Thus, the understanding of the appropriate testing and the diagnostic approaches are crucial for the paediatrician when advising parents regarding the child’s life-long gluten-free diet [15].

## Conclusion

The degree of knowledge of CD (in particular on CD related diseases and CD associated diet needs) among our paediatricians was self-assessed and self-reported in the study. Self-assessment of the degree of knowledge was corroborated by the number of consultations that those FPs reported.

Our findings, however, do not reflect attitude and knowledge of all paediatricians in our Region, as less than a half of them participated in the study. Participation in the study could have been influenced by either lack of knowledge of the disease, or lack of celiac patients. Three paediatricians, who stated not having any celiac patients, completed the questionnaire up to the third question (i.e. how many children do you normally provide care to, and of these how many are celiac patients). Nevertheless, we will take advantage of diffusing the results of the study and the consequent recommendations to all paediatricians in the Region to involve as many as possible of them in future activities aiming to a better management of CD.

Actions aiming to continuous education and emphasizing the importance of including information on CD in medical under and postgraduate trainings are crucial as the disease occurs often with systemic manifestation not involving only gastroenterology.

## Competing interests

The authors declare that they have no competing interests.

## Authors’ contributions

FF conceived and designed the study, analyzed and interpreted data and contributed to draft the paper. MD participated in the design of the study and in the statistical analysis. CV and VA contributed to draw the study protocol and the questionnaire. PR and CT piloted the questionnaire. CP participated in the design of the study and acquisition of data. PR conceived the study, participated in its design and revised the manuscript. PB coordinated the study, interpreted data and revised the manuscript. All authors have given final approval of the version to be published.

## Pre-publication history

The pre-publication history for this paper can be accessed here:

http://www.biomedcentral.com/1471-230X/14/38/prepub
